# Effect of Daikenchuto (TJ-100) on gastrointestinal symptoms following laparoscopic colectomy in patients with colon cancer: study protocol for a randomized controlled trial

**DOI:** 10.1186/s13063-017-2307-7

**Published:** 2017-11-21

**Authors:** Nobuaki Hoshino, Kenji Kawada, Koya Hida, Toshiaki Wada, Ryo Takahashi, Mami Yoshitomi, Yoshiharu Sakai

**Affiliations:** Department of Surgery, Graduate School of Medicine, Kyoto University, 54 Shogoin- Kawara-cho, Sakyo-ku, Kyoto, 606-8507 Japan

**Keywords:** TJ-100, Laparoscopic colectomy, Postoperative gastrointestinal symptoms

## Abstract

**Background:**

Postoperative paralytic ileus can be a difficult complication for both surgeons and patients. Causes and treatments have been discussed for more than two centuries, but have not yet been fully resolved. Daikenchuto (TJ-100, DKT) is a traditional Japanese herbal medicine. Recently, some beneficial mechanisms of DKT to relieve paralytic ileus have been reported. DKT can suppress inflammation, increase intestinal blood flow, and accelerate bowel movements. Therefore, we have designed a randomized controlled trial to investigate the effects of DKT on postoperative gastrointestinal symptoms following laparoscopic colectomy in patients with left-sided colon cancer at a single institution.

**Methods/design:**

As primary endpoints, the following outcomes will be evaluated: (i) grade of abdominal pain determined using the numeric rating scale (NRS), (ii) grade of abdominal distention determined using the NRS, and (iii) quality of life determined using the Gastrointestinal Quality Life Index (GIQLI). As secondary endpoints, the following will be evaluated: (i) postoperative nutritional status (Onodera’s Prognostic Nutritional Index (PNI) and the Controlling Nutritional Status score (CONUT score)), (ii) duration to initial flatus, (iii) duration to initial defecation, (iv) bowel gas volume, (v) character of stool (Bristol Stool Form Scale), (vi) defecation frequency per day, (vii) postoperative complications (Clavien-Dindo classification), (viii) length of postoperative hospital stay, and (ix) metabolites in the stool and blood. This trial is an open-label study, and needs to include 40 patients (20 patients per group) and is expected to span 2 years.

**Discussion:**

To our knowledge, this is the first randomized controlled trial to investigate the effects of DKT on postoperative subjective outcomes (i.e., postoperative quality of life) following laparoscopic colectomy as primary endpoints. Exploratory metabolomics analysis of metabolites in stool and blood will be conducted in this trial, which previously has only been performed in a few human studies. The study aims to guide a future full-scale pragmatic randomized trial to assess the overall effectiveness of DKT to improve the postoperative quality of life following laparoscopic colectomy.

**Trial registration:**

UMIN-CTR (Japan), UMIN000023318. Registered on 25 July 2016.

**Electronic supplementary material:**

The online version of this article (doi:10.1186/s13063-017-2307-7) contains supplementary material, which is available to authorized users.

## Background

Postoperative paralytic ileus can be a difficult complication for both surgeons and patients. Causes and treatments have been discussed for more than two centuries, but have not yet been fully resolved [1]. The incidence of postoperative ileus following colectomy is reported to be at least 10%, and is considered to be inevitable for patients undergoing abdominal surgeries [2]. Patients who suffer from postoperative ileus require additional treatments such as fasting, antibiotics, decompression tubes, or even surgical intervention for suspected bowel strangulation. Owing to this, hospital stays lengthen and medical costs can increase more than 15% [2]. Therefore, the prevention of postoperative ileus is crucial for surgeons.

Daikenchuto (TJ-100, DKT) is a traditional Japanese herbal medicine (Kampo). This medicine is a crude drug extract and consists of four active components including processed ginger, ginseng, Japanese zanthoxylum peel and koi (maltose powder). In Japan, DKT has been clinically used for the treatment of postoperative paralytic ileus [3]. Recently, some beneficial mechanisms of DKT to relieve paralytic ileus have been reported. DKT can suppress inflammation, increase intestinal blood flow, and accelerate bowel movements [4–7]. The anti-inflammatory effect is derived from inhibited activity of cyclooxygenase-2 and upregulation of endogenous adrenomedullin [4, 5]. An increase in intestinal blood flow is due to upregulation of calcitonin gene-related peptide [6]. Accelerated bowel movement is due to adjustment of the contraction and relaxation of the intestine by release of acetylcholine, nitric oxide and other excitatory neurotransmitters [8]. Therefore, DKT is considered to be a potential drug to reduce postoperative paralytic ileus and improve postoperative quality of life for patients with colon cancer.

## Methods/design

### Objective

The aim of this study is to assess the effect of DKT on postoperative gastrointestinal symptoms following laparoscopic colectomy in patients with left-sided colon cancer.

### Study design and setting

This study will be conducted at Kyoto University Hospital in Japan, and the design is an open-label randomized controlled trial. This study is expected to span a period of 2 years. Perioperative procedures in both groups will be performed based on the clinical path for laparoscopic colectomy in Kyoto University Hospital. All patients will receive standard bowel preparation (75 mg sodium picosulfate and 12 mg pursennid) and antibiotic prophylaxis (oral doses of 1 g kanamycin and 750 mg metronidazole), and surgical procedures will be conducted by well-experienced, board-certified laparoscopic colorectal surgeons at our institution. Except for the administration of DKT, treatment protocols including perioperative management and surgical procedures will be congruent among the intervention group (DKT group) and the control group (non-DKT group).

### Endpoints

The following primary endpoints will be evaluated in this clinical trial: (i) grade of abdominal pain determined using the numeric rating scale (NRS), (ii) grade of abdominal distention determined using the NRS, and (iii) quality of life determined using the Gastrointestinal Quality Life Index (GIQLI) [9]. NRS measurements of abdominal pain and distention will be taken prior to surgery and at postoperative days (POD) 1, 4, 7, 14 and 28. GIQLI will be taken before surgery and at POD 14 and 28.

**Fig. 1:**
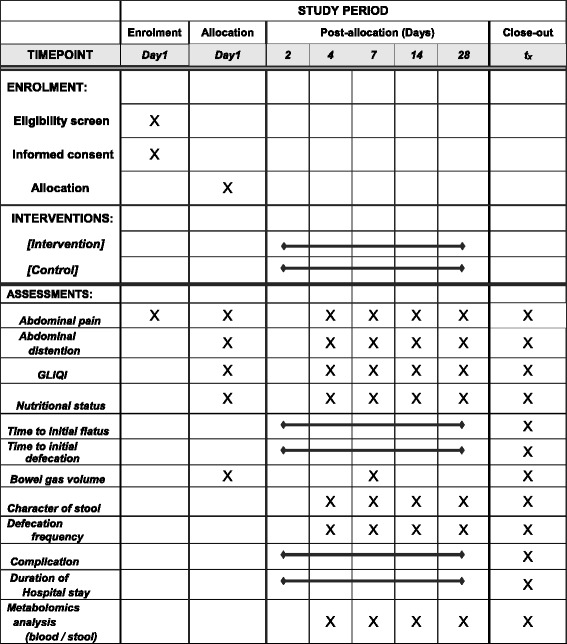
Schedule of enrolment, intervention, and assessments. GIQLI, Gastrointestinal Quality Life Index

The secondary endpoints are (i) postoperative nutritional status (Onodera’s prognostic nutritional index (PNI) [10] and Controlling Nutritional Status score (CONUT score) [11]), (ii) time to initial flatus, (iii) time to initial defecation, (iv) bowel gas volume measured using analysis software, (v) character of stool (Bristol Stool Form Scale [12]), (vi) defecation frequency per day, (vii) postoperative complications (Clavien-Dindo classification [13]), (viii) duration of postoperative hospital stay, and (ix) metabolomics analysis of metabolites in stool and blood using gas chromatography-tandem mass spectrometry (GC/MS/MS) and liquid chromatography-tandem mass spectrometry (LC/MS/MS) (Fig. 1).

### Eligibility criteria

The eligible patients must satisfy the criteria defined below. Potential participants who meet the inclusion criteria will be recruited at outpatient clinics or during preoperative visits before surgery.

Inclusion criteria:

1. Patients with left-sided colon cancer (including recto-sigmoid cancer) who are scheduled to undergo laparoscopic surgery
2. Clinical stage I, II and III
3. Patients who suffer from abdominal pain and distention at POD 1 (NRS score ≥1)
4. The European Cooperative Oncology Group (ECOG) performance status of 2 or less.
5. Patients aged 20 years and older at registration
6. Patients who can take medications orally
7. Written informed consent provided to participate in the study

Exclusion criteria:

1. Patients who have history of abdominal surgery or history of bowel obstruction
2. Patients with concomitant inflammatory bowel disease such as ulcerative colitis and Crohn’s disease
3. Patients with concomitant endometriosis
4. Patients requiring emergency surgery
5. Patients who have been or will be treated by chemotherapy or radiotherapy
6. Patients with severe comorbidity such as cardiac disease, liver disease, pulmonary disease or renal disease
7. Patients who took Japanese herbal medicine (Kampo) up to 4 weeks prior to registration
8. Patients who took gastrointestinal prokinetic drugs, antipsychotic drugs or antidepressant drugs up to 4 weeks prior to registration
9. Patients with a history of a Kampo allergy in other formulations
10. Patients with hepatitis B or C
11. Patients who are unable to take medications orally at POD 1
12. Patients who are unsuitable for study inclusion as determined by the investigator (e.g., those with severe dementia)

### Registration

**Fig. 2:**
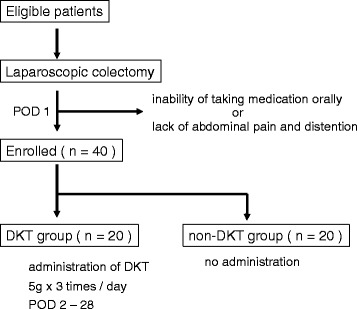
Study flow chart. Investigators obtain informed consent from eligible patients before surgery. Patients are enrolled in the trial, if they can take medicine orally and have abdominal pain and/or distention at postoperative day (POD) 1. Enrolled patients are randomly assigned to either the Daikenchuto (DKT) or non-DKT group

Patients will be enrolled at POD 1. An eligibility report form will be sent to the registration center (APO PLUS STATION Co., Ltd, Tokyo, Japan). Eligible patients will be centrally randomized to either the DKT group or non-DKT group at the registration center using a computer random number generator with the minimization method for TNM stage, tumor location, and age. Information on the necessary follow-up evaluations will then be sent from the registration center. A flow diagram of this study is shown in Fig. 2.

### Treatment methods

#### Intervention group: DKT group

DKT (5 g) will be administered orally three times per day between POD 2 and POD 28. DKT manufactured by Tsumura & Co (Tokyo, Japan) will be used. We will monitor adherence by checking the number of doses of DKT that are not administered.

#### Control group: non-DKT group

No additional medicine will be administered as a comparator.

### Prohibited or limited drugs

Prohibited drugs will be drugs that are known to regulate intestinal movement (erythromycin, mosapride citrate, pantethine, panthenol, prostaglandin F2α), digestive enzyme preparations (diastase, pancreatin), anticholinergic drugs (atropine sulfate, scopolamine butylbromide), cathartic drugs (sodium picosulfate), antidiarrheal drugs (albumin tannate, aluminum silicate, dimethicone), antipsychotic drugs (diazepam, flunitrazepam), lactobacillus preparation (lactomin, lactic acid bacteriae, *Clostridium butyriam*), anti-Parkinsonian drugs, and contrast agent (gastrografin).

Limited drugs will be antiemetic drugs including metoclopramide and domperidone. Rescue drugs will be antidiarrheal drugs (magnesium oxides, sennoside), cathartic drugs (loperamide), and analgesic drugs (loxoprofen). If patients use the limited or rescue drugs, investigators will notate the dose and frequency.

### Criteria for discontinuing the protocol treatment

If grade 3 postoperative adverse effects (Clavien-Dindo classification) are observed, the protocol treatment will be immediately discontinued.

### Data collection

Prospective data about all patients including physical examination, laboratory data, perioperative clinical information and complications will be collected. Personal information including names and chart numbers will not be collected. Only the study code will be collected and managed separately. The collected data will remain confidential until the investigators analyze the data. The final dataset will be managed by the chief investigator (KK). After the completion of the study, the collected data will be encrypted and stored for 10 years and then discarded. Data collection, management, analysis, and interpretation of data will be performed by the registration center (APO PLUS STATION Co., Ltd).

### Sample size determination

The sample size calculation is based on a primary endpoint, GIQLI. The GIQLI questionnaire containing 36 questions each with five response categories. The responses to questions are summed to give a numerical score (0–128 points). A previous report [9] indicates that the mean ± standard deviation (SD) change in the GIQLI score between two measurements before and 2 weeks after laparoscopic cholecystectomy in 194 patients was 17 ± 20.7 points. Therefore, the clinically significant mean difference in the GIQLI score is set at 20 with a SD of 20. Using a power level of 80% and a two-sided significance level of 5%, 32 patients (16 patients in each group) are required to show a difference between the DKT and non-DKT groups. Therefore, we determined that 40 patients (20 patients in each group) are needed to endure 20% dropout of patients from this trial.

### Statistical analysis

All analyses will be performed under the intention-to-treat principle. However, patients will be excluded from the analyses if they are proved to be ineligible after enrollment. Both primary and secondary endpoints will be compared between the DKT group and the non-DKT group. A two-tailed *P* value less than 0.05 is considered statistically significant. For continuous variables, the *t* test will be used when continuous variables follow a normal distribution, whereas the Wilcoxon rank-sum test will be used when continuous variables follow a non-normal distribution. For categorical variables, the chi-square test or Fisher’s exact test will be used according to the cell count.

### Monitoring

This trial is conducted based on Good Clinical Practice. Two people external to trial involvement will check the following at 6 months:

1. Informed consent and the data of primary endpoints for all patients
2. Eligibility criteria and hospital stay duration for a sample of 10 patients

### Study status

This study is currently collecting data and there has not been any publication on the analysis of the data collected to date.

## Discussion

This trial will elucidate the effect of DKT on postoperative gastrointestinal symptoms following laparoscopic colectomy in patients with colon cancer. We mainly focus on two important topics: postoperative quality of life and postoperative metabolites in stool and blood.

Although several trials have been conducted, the clinical effectiveness of DKT is still unclear. In previous randomized controlled trials, all primary endpoints were objective outcomes, such as duration to initial flatus and incidence of postoperative paralytic ileus [14–18]. Although some patients were reported to be improved by DKT administration, there has been no report where any subjective outcomes (i.e., postoperative quality of life) are set up as primary endpoints. Assessment of postoperative quality of life has been conducted as a secondary endpoint in some trials, but there was no significant difference between the intervention group and control group [15–18]. This study is the randomized controlled trial to investigate the effects of DKT on postoperative subjective outcomes (i.e., postoperative quality of life) following laparoscopic colectomy as primary endpoints. Moreover, exploratory metabolomics analysis of metabolites in stool and blood will be conducted in this trial. It was reported that DKT could influence the microbiome of stool through bacterial metabolism in animals’ bowels [19, 20]. However, there is no report to date investigating this in humans. Using metabolomics analysis of metabolites in stool and blood, we plan to examine the effect of the intestinal flora by DKT administration, which can lead to the elucidation of the mechanism of DKT in gastrointestinal symptoms and the discovery of the biomarker candidates.

Therefore, we designed this trial to evaluate the effect of DKT on postoperative quality of life and postoperative metabolites in patients with colon cancer undergoing laparoscopic colectomy. The study aims to guide a future full-scale pragmatic randomized trial to assess the overall effectiveness of DKT to improve the postoperative quality of life following laparoscopic colectomy. Methodological rigor, including random allocation and prospective trial registration is aimed at reducing the risk of bias. As a limitation of this study, the possibility of insufficient sample size, bias in the open-label design, and the possibility of unblinding of the assessors to outcome may result in performance bias.

## Trial status

Recruitment of participants has been ongoing since September 2016.

## Additional file

Additional file 1: SPIRIT 2013 checklist: recommended items to address in a clinical trial protocol and related documents. (DOC 123 kb)

## Abbreviations

CONUT: Controlling nutritional status
DKT: Daikenchuto
GIQLI: Gastrointestinal Quality Life Index
NRS: Numeric rating scale
PNI: Prognostic nutritional index
POD: Postoperative day
SD: Standard deviation
